# Aspects of Austenitization for the Bearing Steel Induction Quenching Design

**DOI:** 10.3390/ma16093523

**Published:** 2023-05-04

**Authors:** Daniela Nachazelova, Jaromir Dlouhy, Petr Motycka, Jakub Kotous

**Affiliations:** COMTES FHT a.s., Prumyslova 995, 334 41 Dobrany, Czech Republic; jaromir.dlouhy@comtesfht.cz (J.D.); petr.motycka@comtesfht.cz (P.M.); jakub.kotous@comtesfht.cz (J.K.)

**Keywords:** carbide, dissolution, austenitization, 100CrMnSi6-4 bearing steel, spheroidization, transformation temperature, induction

## Abstract

The dissolution of carbides during the heating to the quenching temperature has a significant effect on the martensite oversaturation and the resulting mechanical properties. The kinetics of dissolution can be influenced by various external factors. This work deals with monitoring the carbide dissolution utilizing dilatometer analysis. The austenitization of 100CrMnSi6-4 bearing steel in two initial states was compared—after accelerated spheroidization annealing and conventional soft annealing. The main objective was to determine the amount of undissolved cementite during austenitization in the temperature range where only austenite and cementite are present in the structure. The austenitization temperature determines the degree of cementite dissolution and, consequently, the carbon content in austenite and thus the final properties after quenching. The cementite dissolution was quantified from dilatometric curves and image analysis.

## 1. Introduction

Bearing steels are used in the hardened state, i.e., after quenching and tempering. The final properties of the tempered martensite are by a large extent determined by the carbon content in the martensite. Hypereutectoid steels, such as the 100CrMnSi6-4 and similar grades, contain roughly 1 wt.% carbon. This amount is too high to obtain martensite of suitable properties if dissolved in the austenite completely upon austenitization before quenching [1]. Therefore, only partial carbide dissolution is necessary during austenitization. This requires setting the austenitization parameters accordingly [2,3]. Heating in a furnace is usually performed with a sufficient hold at a temperature that can ensure a close-to-equilibrium state. It is possible to use the equilibrium diagram or thermodynamic equilibrium calculation in this case. This approach usually cannot be relied on in the case of induction hardening. It consists of induction heating and subsequent quenching. The material is heated at a high rate (up to 100 °C/s), usually with no hold at the quenching temperature. This treatment does not allow for the equilibrium state to be established. Therefore, austenitization and carbide dissolution kinetics, the heating rate and the type of initial microstructure influence the setting of the quenching temperature required for the desired carbide dissolution stage [4,5]. 

Comprehensive approaches to the theory of induction heating are available in the literature [6]. The heating itself can be modeled really accurately for the material of known physical properties. Close attention is paid to the induction heating equipment and heating measurement [7,8,9]. However, not so much space is devoted to the setting up of the quenching temperature during induction heating. The quenching temperature directly determines the stage of carbide dissolution and thus the amount of carbon in austenite upon quenching. The setting of this critical parameter is covered in the literature by a rough estimation for given steel ([9]-page 191), with a note that the differences in required austenitization temperature can vary by more than 100 °C due to differences in the heating rate and microstructure. Thus, the quenching temperature setting is left to the trial-and-error method.

Such an approach can be pretty straightforward in the case of the treatment of small and cheap parts, where several or even several dozen test pieces can be easily heated, quenched and measured to see whether they meet the required properties. However, this trial run of the technology can be unfavorable or even unacceptable when the treated parts are large, expensive or simply available in a limited number.

Trial-and-error parameter setting is also problematic due to the fact that the hardness is usually the only required and actually measured material property after the induction quenching. A high hardness is normally required in the case of high-carbon steels, such as the bearing steel 100CrMnSi6-4, 63 HRC (Rockwell hardness—see Table 1) and more. In this hardness interval, there is no clear dependence of hardness on carbon content in parent austenite (i.e., on quenching temperature) as more carbon in austenite does not cause a clear hardness increase, like for lower carbon content [10]. Thus, it is possible to obtain “correct” hardness by the quenching of overheated high-carbon austenite, resulting in coarse high-carbon martensite and a high content of residual austenite. This has an inevitably detrimental effect on the mechanical properties of the final product and can be unnoticed if no metallography analysis of the quenched part is executed.

This article offers an alternative to the trial-and-error method for quenching temperature determination. It describes the use of dilatometry measurement and metallography analysis to determine the quenching temperature based on the demanded hardness. This approach is less demanding on the experimental equipment than, for example, in situ X-ray diffraction analysis or thermomechanical simulation [4,11]. The influence of the carbide size and heating rate on final hardness was examined for widely used bearing steel 100CrMnSi6-4.

The carbide size and shape gain importance when non-equilibrium states of carbide dissolution are considered [12,13,14]. Finer carbides dissolve more rapidly upon austenitization and are also able to reach finer and harder martensite, as documented in previous work [14,15,16]. Bearing steels are usually soft annealed to form the microstructure of spheroidized carbides in the ferrite matrix with good machinability and formability. The soft annealing (SA) [17], which can last up to tens of hours [18], ensures carbide spheroidization in a ferrite matrix [19]. Much finer spheroidized carbides can be produced by accelerated carbide spheroidization and refinement (ASR) [20]. The microstructures obtained by SA and ASR were used as initial structures for this experimental induction quenching. They are morphologically identical (spherical carbides in a ferrite matrix) and just differ in carbides size.

The carbide dissolution was monitored by sample dilatation until full austenitization at various heating rates. Then, the quenching temperature was set for each heating rate according to the arbitrarily chosen degree of carbide dissolution determined from the dilatometer curve. The undissolved carbide content was determined by image analysis of the microstructure. It was proved that the dilatometer record can be used for setting the quenching temperature to achieve a certain degree of carbide dissolution. Moreover, the quantification of undissolved carbide content enables the setting of the quenching temperature to the desired amount of carbon dissolved into the austenite and thus the desired hardness of the material after quenching. 

The carbon content in austenite was also measured indirectly by the measurement of the martensite start (M_s_) temperature. Generally, when more carbon is in the austenite the M_s_ is decreasing [21]. 

Dilatometer and metallography analysis can be used for setting the parameters of induction heating in cases when an extensive run of test trials is not possible due to costs or due to a lack of spare pieces for the test trials.

## 2. Materials and Methods

### 2.1. Experimental Material

The experimental material was the 100CrMnSi6-4 bearing steel grade with the chemical composition given in Table 2. The material was supplied in the form of hot-rolled 21 mm diameter bars. The as-received microstructure consisted of lamellar pearlite with a small amount of secondary cementite. The hardness of the as-received material was 383 HV10 [22].

### 2.2. Spheroidization Annealing

The as-received state of the lamellar pearlite was further processed by two annealing methods to obtain a structure with different sizes of globular carbides. The samples were 16 mm in diameter and 100 mm in length. The finer globular cementite was prepared by accelerated spheroidization and refinement (ASR) annealing using induction heating in a medium-frequency source (f_max_ = 17 kHz) with a maximum power of 25 kW. The ASR process consisted of induction heating of 15 °C/s to 800 °C with a 15 s residence time and air cooling to 680 °C. This cycle was repeated twice more, followed by the cooling of the material in air to ambient temperature [22]. 

**Figure 1:**
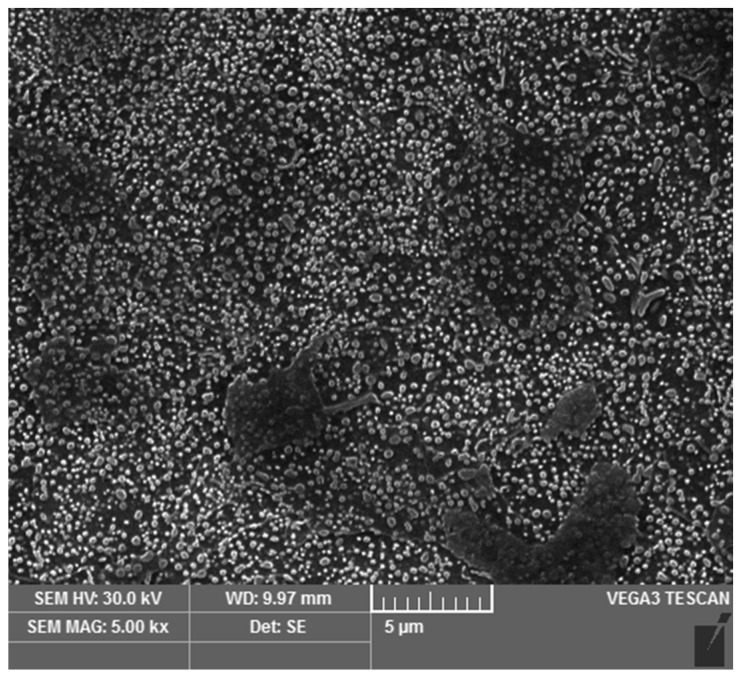
Microstructure after ASR (accelerated spheroidization and refinement) process.

**Figure 2:**
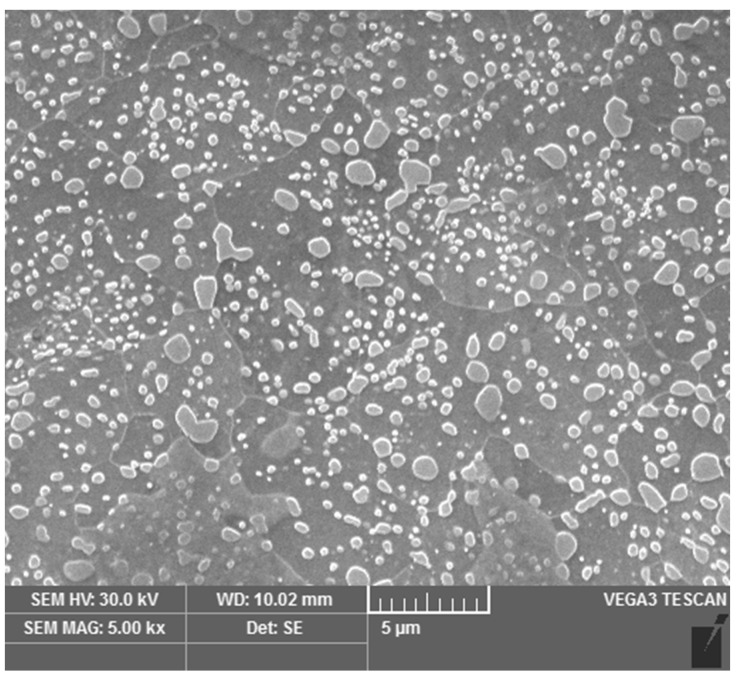
Microstructure after conventional soft annealing (SA).

The resulting microstructure was a fine spheroidized cementite (Figure 1). Coarser globular cementite (Figure 2) was prepared by conventional soft annealing (SA) in an atmospheric furnace. The conventional soft annealing regime consisted of a 15 hr temperature hold at 810 °C and controlled cooling in the furnace at 13 °C/hr. These two spheroidized microstructures were used as initial states for the experiment.

### 2.3. Dilatometric Experiments for the Monitoring of Carbides Dissolution

**Figure 3:**
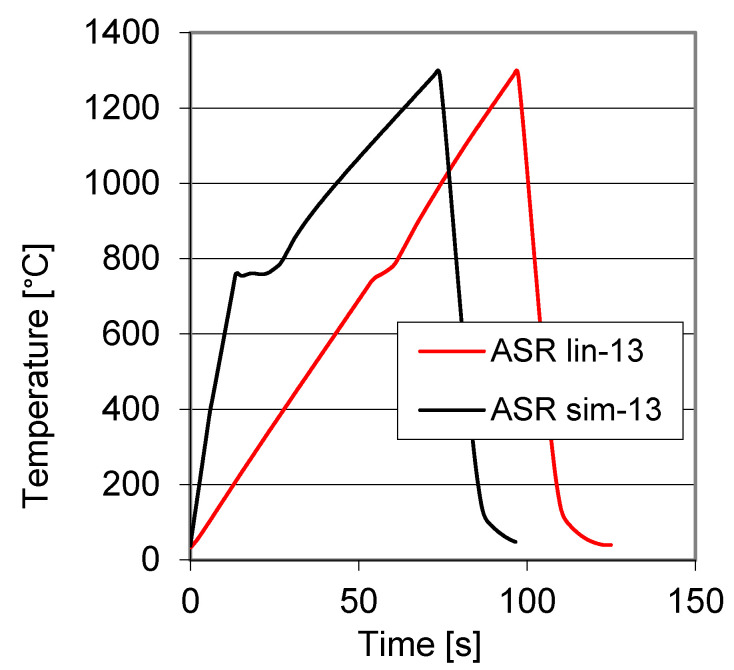
Dilatometric curves from linear heating (ASR lin-13) and the simulation of real induction heating in a medium-frequency converter (ASR sim-13).

The cylinders with a diameter of 4 mm and a length of 10 mm were extracted from 100CrMnSi6-4 steel samples in two initial states: after soft annealing (SA) and ASR treatment. The specimens were treated in the quenching dilatometer L78RITA (Rapid Inductive Thermal Analysis, producer Linseis, Selb, Germany). The dilatometer allows the specimen temperature to be controlled precisely and with a very short response time during heating and cooling at a rate of up to 200 °C/s. The specimens were heated by the induction coil and cooled by flowing helium. They were heated by the linear regime with a constant heating rate (labelled as “lin”) as well as by the regime simulating heating by an industrial inductor with a fixed power output (labelled as “sim”). Constant heating output results roughly in linear heating when the thermal capacity is constant; however, the heating stops when a transformation with a latent heat proceeds (such as the austenitization). This creates a delay in heating. The simulation regime was obtained from the induction heating of a 16 mm diameter, 100 mm long rod with a welded thermocouple. It was performed in a medium-frequency converter (f_max_ = 17 kHz) with a maximum power of 25 kW. According to measured data, heating was then simulated in a quenching dilatometer (Figure 3). This simulation replicates the real processing process, thus minimizing the influence of factors commonly used in linear dilatometer heating simulations.

The samples were heated by heating rates 6, 13 and 22 °C/s in the austenitic state. An explanation of the marking of the samples is given in two examples below: 

ASR lin-6: initial state after accelerated ASR processing, linearly heated to the austenitizing temperature at 6 °C/s.

SA sim-13: initial state after conventional soft annealing, simulated heating to the austenitizing temperature at approx. 13 °C/s according to the heating of the bar in medium-frequency induction equipment.

### 2.4. Image Analysis

The amounts of carbides in the structures were determined by image analysis. The dilatometric specimens were cut longitudinally and the section was mechanically ground and polished with the last mechanical polishing step being 1 µm diamond suspension. The final polishing step was chemo-mechanical polishing by OP-S colloidal silica suspension (provided by Struers). Its average grain size is 50 nm and it causes a slight etching of the steel surface during polishing. OP-S polishing was carried out for 3 min. 

The specimens were observed in a scanning electron microscope (SEM) with an acceleration voltage of 5 kV. Ten images were captured from random locations on the sample. The carbide content was evaluated by the point method [23]. Ten randomly chosen image fields were used for each sample, and a grid of 19 × 14 points was evaluated for each field.

## 3. Results and Discussion

**Figure 4:**
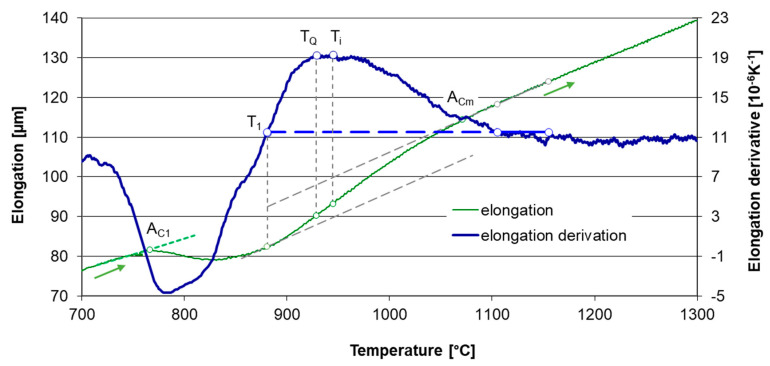
The elongation of the sample ASR-lin-6 and its temperature derivative. A_1_ is the starting point of transformation, T_1_ is the point at which the elongation derivative equals to the derivative for homogeneous austenite, T_i_ is the point of the maximum elongation derivative. T_Q_ is the quenching temperature for the metallographic sample for the evaluation of the absolute amount of carbides.

The first group of dilatometric specimens was heated up to 1300 °C to determine the austenite homogenization temperature interval. The dilatometer curves were recorded for the purpose of austenite formation and homogenization tracking (Figure 4). A_C1_ and A_Cm_ transformation temperatures were determined from the dilatometer curves (Table 3). The specimens in the second group were heated at the same heating rates. The heating was interrupted by sudden quenching from various temperatures to capture carbide dissolution into austenite in a particular stage. These specimens were used for metallographic quantitative analysis of carbides content. The quenching temperatures T_Q_ were determined from the first specimen group.

### 3.1. Determination of Transformation Temperatures

Heating to 1300 °C was performed to obtain dilatometer curves through the whole austenitization interval up to the state of homogeneous austenite. The heating rates were 6, 13 and 22 °C/s and they were monitored mainly over temperature A_c1_. In addition to linear heating at a certain rate, which is commonly used for heating during dilatometric measurements, a simulation of real induction heating was performed (Figure 3). 

**Figure 5:**
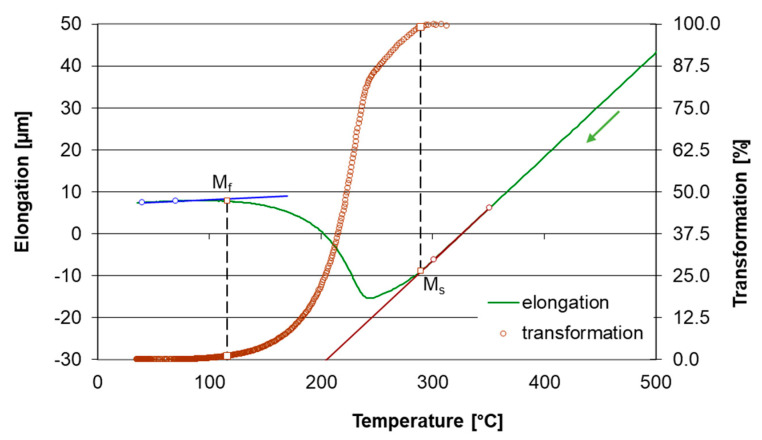
The illustration of the use of lever rule to determine M_s_ temperature.

The total specimen elongation at heating is the sum of the thermal expansion of all phases, decrease in length due to austenite forming and increase in length due to carbide dissolution. Temperature intervals for phase transformation and carbide dissolution certainly overlap. The dilatometric effect of carbide dissolution prevails over the effect of austenite formation above temperature T_1_ shown in Figure 4. The transformation temperatures A_c1_ and M_s_ were determined by the lever rule as 1% elongation deviation from the linear trend before transformation to the linear trend after transformation. The temperature A_Cm_ was estimated as the point where the elongation deviation is only 1% from the trend for homogeneous austenite compared to its maximum value at point T_1_ shown in Figure 4. An example of the use of the lever rule for determining M_s_ temperature is given in Figure 5.

### 3.2. Carbide Dissolution Quantification

The specimens for the metallographic determination of the absolute amount of carbides were heated in the same way as samples for determining the austenite homogenization interval, but they were quenched by a cooling rate of 200 °C/s after reaching a temperature at which a significant occurrence of carbides in the structure simultaneously with full austenitization was ensured. This point was found at three-quarters of the band between the linearized trends of T_1_ and T_i_ according to the heating rate (Figure 4). It was chosen arbitrarily, the only necessity being the full austenitization of the ferritic matrix. Thus, all specimens from the second group were quenched from different temperatures T_Q_ but at the same point regarding the general shape of the dilatometer curve.

The following Table 3 shows transformation temperatures that were evaluated from the individual dilatometric curves (for examples, see Figure 4 and Figure 5), the hardness and the amount of undissolved carbides.

**Figure 6:**
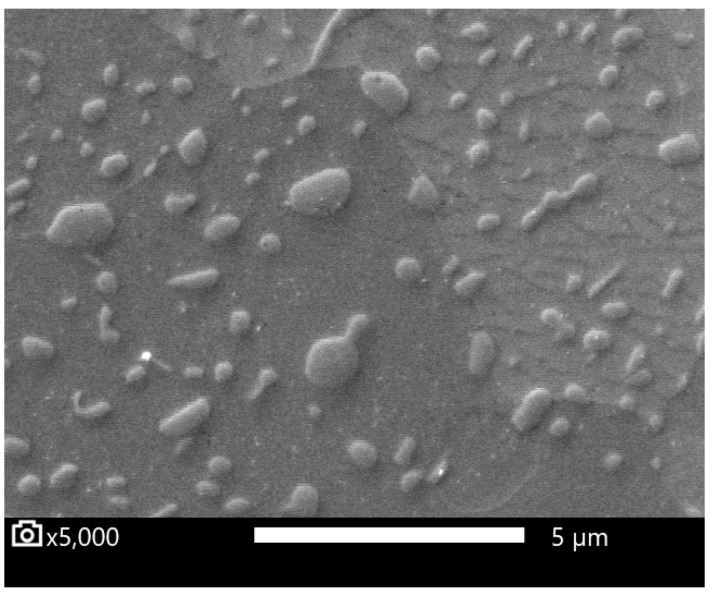
Specimen SA—initial state. Globular carbides in the ferrite matrix. Polished specimen.

**Figure 7:**
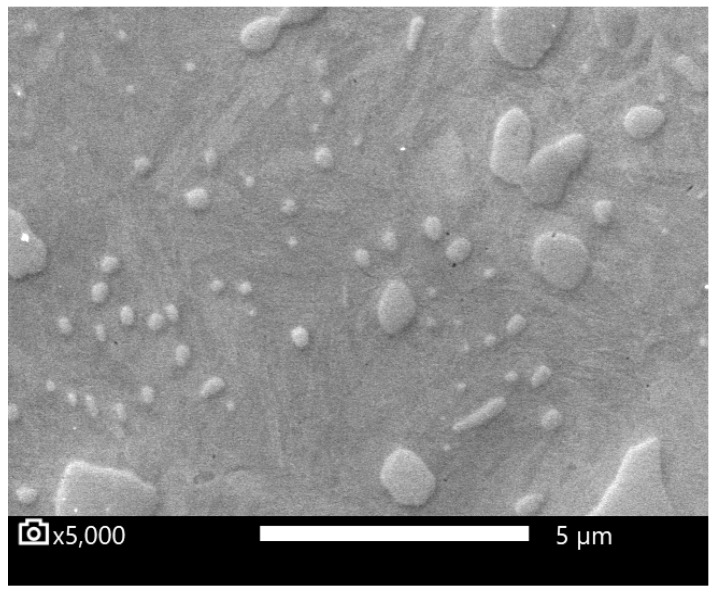
Specimen SA-sim-13, quenched from T_Q_ temperature. Undissolved carbides in a martensitic matrix. Polished specimen.

Metallograph sections from the quenched specimens were prepared, as well as sections from the initial states, ASR- and SA-treated steel. The volume content of cementite was measured for these specimens by image analysis. SEM images showed a significant dissolution of the finer carbides after heating to the T_Q_ temperature (see Figure 6 and Figure 7). 

### 3.3. Carbide Content Quantification in Dilatometer Curve

**Figure 8:**
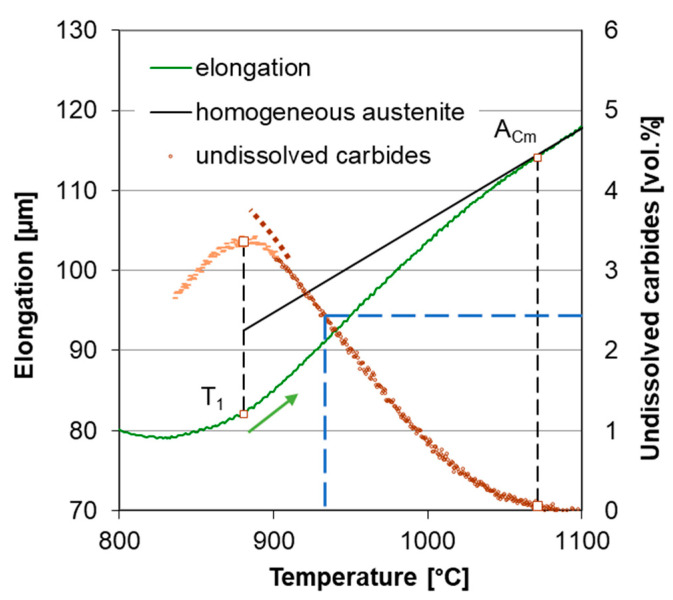
Dilatometric curve of ASR-lin-6 (green line); amount of undissolved carbides (brown line). T_Q_ for this sample was 923 °C, undissolved carbide content at T_Q_ was 2.5 vol.% (see Table 3).

**Figure 9:**
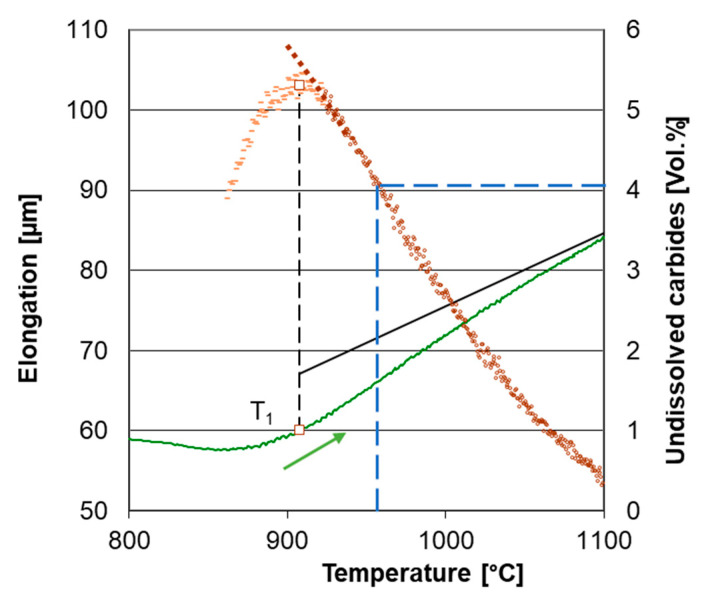
Dilatometric curve of SA-lin-6 (green line); amount of undissolved carbides (brown line). T_Q_ for this sample was 954 °C; undissolved carbide content at T_Q_ was 4.1 vol.% (see Table 3).

Image analysis gave a value of carbide content for one particular temperature during the specimen heating. This enabled the relation of the dilatation of the sample to the amount of undissolved carbides, as is shown in Figure 8 and Figure 9. The representation of dilatometer curves as a direct measurement of carbide content is valid only with an assumption that no process other than carbide dissolution determines specimen dilation. This can be assumed only above temperature T_1_, where the specimen’s length monotonously decreased, as expected during carbide dissolution. Other phenomena obviously contributed to the specimen dilation at lower temperatures—mainly the phase transformation of the matrix. 

### 3.4. Carbide Dissolution and Final Hardness

**Figure 10:**
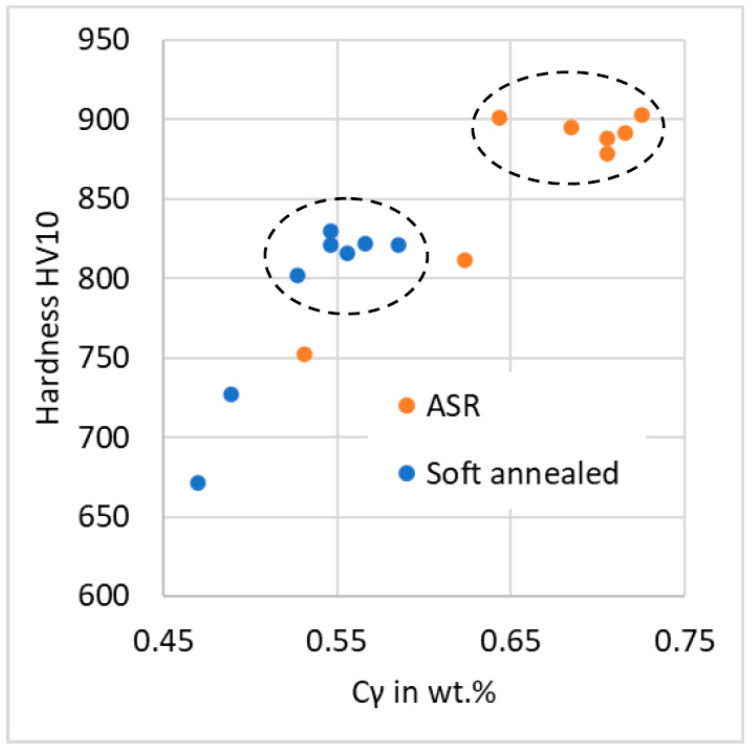
The plot of “Hardness-C_γ_” relationship. Encircled groups of points represent T_Q_ specimens, heated by both linear and simulated regimes. The points out of the circles represent specimens quenched at temperatures T_Q_—30°C and T_Q_—60 °C.

The carbon is distributed between the austenite matrix and undissolved carbides during the austenitization. The ratio of the initial volume of carbides and volume of undissolved carbides represents the distribution of carbon between the austenite and the carbides. Thus, the carbon content in austenite C_γ_ (in wt.%) was computed from the undissolved cementite volume as follows:

(1) $$C_{\gamma }=0.94\;\left(1-\frac{{CEM}_{Q}}{{CEM}_{init}}\right)$$

where 0.94 is the total amount of carbon in steel in wt.% (see Table 2), *CEM_Q_* is the volume of carbides in the quenched sample and *CEM_init_* is the volume of carbides in an initial microstructure. The dependence of specimen hardness and C_γ_ can be plotted for all quenched specimens (see Figure 10). All specimens quenched from the T_Q_ temperature were grouped together in two distinctive groups for ASR and SA initial state. This points to two things. First, the stage of carbide dissolution (and the resulting value of C_γ_) was quite precisely defined by setting T_Q_ according to the dilatometer curve. Second, the initial state of the material strongly influences the carbide dissolution rate. Based on these results, two additional specimens were heated by the linear regime at the rate of 22 °C/s and quenched from temperatures 30 °C and 60 °C below T_Q_. They reached both lower hardness and lower carbide dissolution, as can be expected. These specimens showed the hardness–C_γ_ relationship, which follows the famous graph published by Krauss [10]. 

To set the quenching temperature for desired hardness, one has to follow these steps:

1. Obtain C_γ_ from Figure 10 for desired hardness;
2. Calculate the amount of undissolved carbides *CEM_Q_* from Equation (1);
3. Determine the quenching temperature from the dilatometer curve recorded at the corresponding heating rate of the appropriate initial microstructure (e.g., Figure 9).

**Figure 11:**
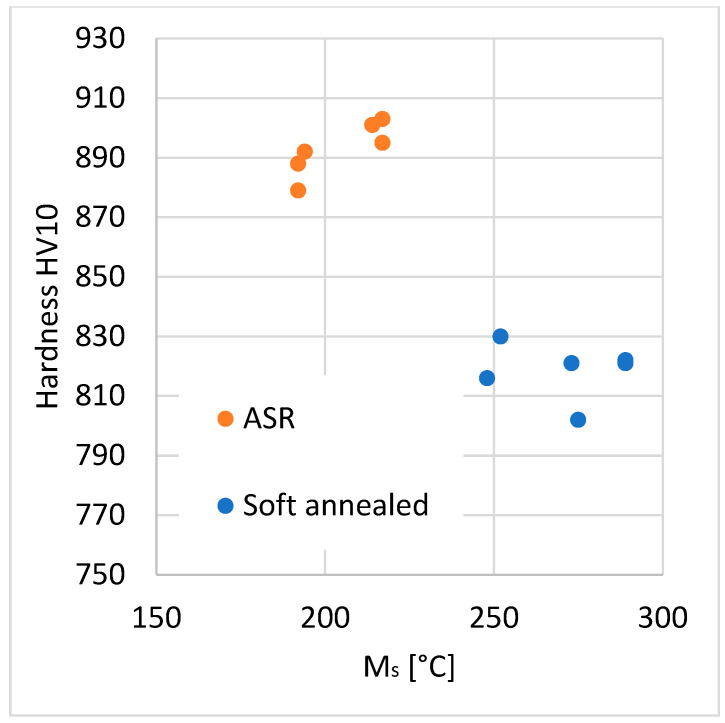
The plot of “Hardness-M_s_” relationship.

A similar plot was drawn for hardness–M_s_ temperature dependence (Figure 11). M_s_ temperature can be used similarly for carbide dissolution quantification. However, it depends on the C_γ_ and also on the prior austenite grain size [1]. Thus, metallography analysis is still necessary for the precise assessment of C_γ_ based on the M_s_ temperature. A rough estimate of C_γ_ can still be made, especially as just a quick check of whether the steel was overheated and whether too much carbon dissolved into austenite.

The spread of points representing specimens quenched from the T_Q_ temperature is noticeably larger in the value of C_γ_ than in the measured hardness. This can be attributed to the hardness measurement being more precise than quantitative image analysis of undissolved carbides. The main obstacle in the image analysis was presumably the low absolute content of undissolved carbides, leading to the large scatter of results.

The type of heating regime—linear or simulated industrial heating—did not affect the results in terms of carbide dissolution and hardness. This confirms that linear heating regimes can be used for predictions of the carbide dissolution and final hardness of the real industrial heating process. The real heating process with a fixed power output has a significantly higher heating rate when the material is paramagnetic (below the A_c1_ temperature) and also a much more pronounced delay in heating at the A_c1_ temperature. These differences do not change the resulting microstructure and properties as long as the heating rate in the austenitic state is the same as for the linear regime.

## 4. Conclusions

1. Dilatometer analysis can be utilized as a tool for setting parameters of induction heating. It was proved that the amount of dissolved carbides in hypereutectoid steel 100CrMnSi6-4 is represented in the dilatometric curve. The dissolution stage can be attributed to a certain point of the curve based on its general shape. Thus, it is possible to determine the quenching temperature on the actual carbon content in the austenite, regardless of the heating rate.
2. The initial structure proved to have a strong impact on the carbide dissolution rate and resulting hardness. The microstructure of the austenitized steel has to be taken into account, rather than only the nominal chemical composition.
3. It is possible to relate the dilatometer curve to the resulting hardness by hardness measurement and the quantification of the undissolved carbide content for specimens with interrupted heating. This can be used for the setting of an appropriate quenching temperature at the given heating rate to reach the required hardness.
4. Linear heating rates normally used in dilatometer measurements can be used as a simulation of real heating regimes by industrial induction devices without any significant result distortion.

## Figures and Tables

**Table 1:** List of symbols and abbreviations.

A_C1_	The start temperature of austenite formation during heating
A_Cm_	The temperature of the last cementite dissolution during heating
ASR	Accelerated carbide spheroidization and refinement
CEM_init_	The volume of carbides in an initial microstructure
CEM_Q_	The volume of carbides in the quenched sample
C_γ_	Carbon content in austenite
HRC	Rockwell hardness, C-scale
HV	Vickers hardness
M_s_	Martensite start temperature during quenching
SA	Soft annealing
SEM	Scanning electron microscope
T_1_	The point at which the elongation derivative equals to the derivative for homogeneous austenite
T_i_	The point of maximum elongation derivative
T_Q_	The quenching temperature

**Table 2:** Chemical composition of 100CrMnSi6-4 bearing steel, wt.%.

C	Si	Mn	P	S	Cr	Ni	Al	Cu
0.94	0.65	1.16	0.014	0.012	1.54	0.03	0.03	0.02

**Table 3:** Results of dilatometric measurement, image analysis and hardness measurement.

Heating to 1300 °C				Heating Interrupted by Quenching at T_Q_			
	Heating Rate (°C/s)	Transformation Temperatures A_C1_ (°C) A_Cm_ (°C)		Quenching Temperature T_Q_ (°C)	Martensite Start Temperature M_s_ (°C)	Hardness HV10	Undissolved Carbides (vol.%)
ASR-lin-6	6	761	1076	923	217	895	2.5
ASR-sim-6	6	748	1052	923	214	901	2.9
ASR-lin-13	13	761	1110	943	194	892	2.2
ASR-sim-13	13	753	1101	943	192	888	2.3
ASR-lin-22	22	764	1152	967	192	879	2.3
ASR-sim-22	22	768	1127	967	217	903	2.1
SA-lin-6	6	773	1151	954	252	830	4.1
SA-sim-6	6	762	1084	954	289	822	3.9
SA-lin-13	13	776	1167	973	289	821	3.7
SA-sim-13	13	775	1124	973	275	802	4.3
SA-lin-22	22	785	1174	997	248	816	4.0
SA-sim-22	22	783	1143	997	273	821	4.1

## Data Availability

The data used in this article are part of the ongoing experimental program and can not be shared.
